# Art, Science, and Life’s Enigmas

**DOI:** 10.3201/eid1208.AC1208

**Published:** 2006-08

**Authors:** Polyxeni Potter

**Affiliations:** * Centers for Disease Control and Prevention, Atlanta, Georgia, USA

**Keywords:** art, science, life’s enigmas, Leonardo da Vinci, Andrea Del Verrocchio, Emanuele Repetti

**Figure:**
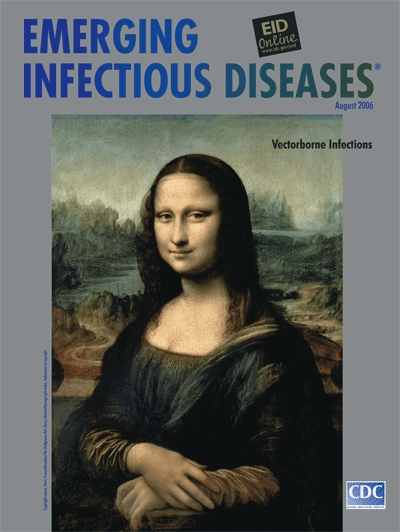
Leonardo da Vinci (1452–1519). Mona Lisa (c.1503–1506). Oil on panel (77 cm × 53 cm). Copyright Louvre, Paris, France/Giraudon/The Bridgeman Art Library. Nationality/copyright status: Italian/out of copyright

Vinci, a small town near Florence, Italy, dates back to Roman times, when it was inhabited by the Etruscans. "But this town is even more renowned for having given its name to the famous Leonardo da Vinci, who, in any discipline of science and art he dedicated himself to, surpassed all his contemporaries" wrote Emanuele Repetti in his geographic dictionary of Tuscany (*1*). In the modern sense, during his lifetime, the great Leonardo had no surname—"da Vinci" means "from Vinci."

Born out of wedlock to a notary-craftsman and a peasant woman, Leonardo was nonetheless well educated in Florence. At this cultural center, home of the Medici, he was apprenticed to sculptor and painter Andrea Del Verrocchio (*2*). "Marvelous and divine, indeed, was Lionardo the son of ser Piero da Vinci" said writer and painter Giorgio Vasari in his Lives of the Artists. During his apprenticeship, charged with painting an angel in Verrocchio's The Baptism of Christ, he painted a face so divine that Verrocchio never touched colors again "angry that a boy should know more than he" (*3*).

"He is a poor pupil who does not surpass his master," Leonardo noted, when his mathematical knowledge exceeded his tutor's (*4*). Botanist, architect, civil and military engineer, town planner, hydrologist, cartographer Leonardo anticipated, 500 years ago, the scientific discoveries of our time. "He made models of mills and presses, and machines to be worked by water, and designs for tunneling through mountains, and levers and cranes for raising great weights, so that it seemed that his brain never ceased inventing; and many of these drawings are still scattered about" (*3*). Though his radical notions (aviation, military hardware, mechanical calculation) escaped his contemporaries, his genius was widely acknowledged in his lifetime. Yet, much of his work has been lost to his flamboyance. Many projects were unfinished or obscured by secrecy and cryptic records. He wrote backwards with the left hand, so notes meant for him alone could be read only in the mirror. Only a dozen or so paintings survive.

At age 30, handsome and gifted, a musician who improvised verses on a lute of his own invention, Leonardo owned a studio in Milan and had several apprentices. He completed his first large painting, Virgin of the Rocks, and the masterpiece Last Supper. Then, he went to Venice and back to Florence to work as military engineer. He traveled to Mantua and Rome, where Raphael and Michelangelo worked, then to Pavia and Bologna. Finally, he left Italy for France and the court of humanist King Francis I. Though he painted little in France, he brought with him some of his great works, including Mona Lisa, which remained there after his death in the king's arms at age 67.

Art and science were aligned harmoniously in Leonardo. Art was guided by science, and science was expressed through art. His studies and experiments, begun in Verrocchio's workshop and recorded in copious notebooks, are masterfully illustrated. To paint better, he studied anatomy, dissecting human bodies and drawing them in detail. His work with optics, especially prisms, which anticipated Newton's, refined his rendition of light and shadow. His figures showing insertion of the muscles and their movements are still admired by anatomists. "It is true that decorum should be observed," he believed, "that is, movements should announce the motion of the mind of the one who is moving" (*4*).

"Lionardo was so pleased whenever he saw a strange head or beard or hair of unusual appearance that he would follow such a person a whole day, and so learn him by heart, that when he reached home he could draw him as if he were present" (*3*). Guided less by his extraordinary talent and more by meticulous technique, he shunned tradition and theory. In the most ubiquitous portrait of all time, Mona Lisa, which graces museums, dormitories, billboards, wine bottles, and now the cover of Emerging Infectious Diseases, the artist paid lip service to the formal vocabulary of Florentine tradition: a half-length figure, turned almost directly toward the viewer, beauty emanating from inner virtue.

Then, he positioned the figure up front to increase drama and intensity. Breaking with tradition, he painted a landscaped background, spatial depth. Instead of outlining the portrait, he merged it with surroundings. Perfecting sfumato, a technique described in antiquity by Pliny, he created an imperceptible transition between light and dark and sometimes between colors, "smoking" harsh edges with brushstrokes invisible to the naked eye. Cognizant of the way light fell on curved surfaces, he used layers of transparent color to capture it on gauzy veil or skin. The result was ethereal, magical, a glow that transformed portraiture for the ages, demanding not just likeness but the embodiment of spirit.

"As art may imitate nature, she does not appear to be painted, but truly of flesh and blood. On looking closely at the pit of her throat, one could swear that the pulses were beating," wrote Vasari (*3*). Mona Lisa, he continued, was painted for Florentine silk merchant Francesco del Giocondo, who commissioned it for his wife Lisa Gherandini, Mona (Madame) Lisa del Giocondo (La Gioconda), to mark the birth of their second son. Leonardo worked on the painting for several years and parted with it only at death.

"Executed in a manner well calculated to astonish all who behold her," the portrait was prominently displayed, admired, and widely reproduced (*3*). Raphael created a series of portraits with a striking resemblance, and among others, dadaist Marcel Duchamp and surrealist Salvador Dalí produced their mock interpretations. Yet, "those who put the moustache on Mona Lisa," wrote contemporary artist Barnett Newman, "are not attacking it or art, but Leonardo da Vinci the man. What irritates them is that this man with half a dozen pictures has this great name in history, whereas, they, with their large oeuvre, aren't sure" (*4*).

"Mona Lisa being most beautiful, he used while he was painting her, to have men to sing and play to her and buffoons to amuse her, to take away that look of melancholy which is so often seen in portraits; and in this of Lionardo's there is a peaceful smile more divine than human" (*3*). Much has been speculated about the smile, about the painting, about Leonardo. While Vasari was acquainted with the Giocondo family, he did not write his anecdotal biography until more than 30 years after Leonardo's death, and competing accounts of Mona Lisa's origin and identity abound. If the portrait was commissioned, why was it never delivered to its patron? Leonardo himself left scant evidence of his own opinions and ideas and only one definitive self-portrait in red chalk, a venerable face carved by time, framed by flowing hair and beard.

Some attribute the uncanny perfection of Mona Lisa to Leonardo's scientific observation, mathematical instinct, unparalleled skill, and the harmony of the composition (*5*). Others take different paths: "The elusive quality of Mona Lisa's smile…is almost entirely in low spatial frequencies, and so is seen best by your peripheral vision" (6).

The subtle smile, reminiscent of archaic funerary statues (kouroi, korai), the languid eyes, the puzzling backdrop of nature, the intricate loops of the neckline, the calm hands, even the absence of visible facial hair (eyebrows and eyelashes were not the style) add to the mysterious, semi-abstract quality of the face.

The enigma of Leonardo's creation and the intrigue surrounding its origin, identity, and meaning can only be a metaphor for his own life and ideas and, by extension, ours. An archetype of the Renaissance, this man who would and could do everything must have peered inside himself for answers he had sought far afield. And just as he dissected and outlined the physical body, he sought to find and paint the spirit. Always the scientist, he knew that a portrait alone, no matter how exacting, would not do. Rejecting the flat background of tradition, he added the landscape. Part dream, part romantic reality, it provided perspective and connected the figure to the world, adding to the enigma and possibly holding the definitive interpretation.

The puzzles of our era, unknown pathogens, many of them vectorborne, emerging biological threats, ecologic disasters, antimicrobial drug resistance can also benefit from meticulous observation, accurate recording, added perspective, and the interdisciplinary approach to knowledge. Just as with Leonardo, the art is in the science.
